# Metastasis of *in situ* solid papillary carcinoma of the breast to the lungs:a rare case report

**DOI:** 10.3389/fonc.2025.1630957

**Published:** 2025-10-08

**Authors:** Jing Zhou, Minmin Wu, Xiaoli Huang, Keyu Liu, Jingjing Xu

**Affiliations:** Department of Pathology, Shanghai Public Health Clinical Center, Fudan University, Shanghai, China

**Keywords:** breast, solid papillary carcinoma, infiltrative solid papillary carcinoma, metastasis, surgery

## Abstract

Solid papillary carcinoma (SPC) of the breast is considered a special subtype of ductal carcinoma *in situ* (DCIS), characterized by distinctive histological features and growth patterns. Although SPC itself is not very common, it is particularly noteworthy that distant metastasis from its non-invasive form is highly uncommon. This contrasts sharply with infiltrative solid papillary carcinoma (ISPC), which lacks the typical myoepithelial cell layer seen in SPC and displays a map-like infiltrative growth pattern, often involving adipose and fibrous stromal infiltration. This study reports a case of a patient who was diagnosed with *in situ* SPC following a total mastectomy, and subsequently developed pulmonary metastatic ISPC five years later. The case supports the concept of a morphological continuum from *in situ* SPC to invasive carcinoma and highlights that even non-invasive SPC may possess the distant metastasis, albeit rarely. These findings provide new insights into the biological behavior of this rare breast cancer subtype.

## Introduction

Solid Papillary Carcinoma (SPC) is a rare form of papillary lesion of the breast, predominantly occurring in postmenopausal women, and accounts for less than 1% of all breast carcinomas. SPC is characterized by a solid papillary growth pattern with a fibrovascular core. It typically presents as a low-grade tumor with tumor cells of low to intermediate nuclear grade and is frequently associated with neuroendocrine differentiation, exhibiting distinct clinicopathological features (1). According to the 2019 World Health Organization (WHO) classification of breast tumors, the pathological subtypes of Solid Papillary Carcinoma (SPC) are divided into:1. *In situ* solid papillary carcinoma; 2. Solid papillary carcinoma with invasive carcinoma (2). *In situ* SPC is considered a special subtype of ductal carcinoma *in situ*, which is usually asymptomatic though it can have atypical presentation (3). *In situ* may be accompanied by the presence of myoepithelial cells. When myoepithelial cells are completely absent, the lesion is referred to as an expansile or pushing type of invasion, which typically does not metastasize (4, 5). Solid papillary carcinoma with invasive carcinoma refers to the presence of invasive components, such as mucinous carcinoma, invasive ductal carcinoma, or lobular carcinoma, arising within a background of *in situ* SPC. In addition, a distinct subtype known as infiltrative solid papillary carcinoma (ISPC) has been described. Histologically, ISPC is characterized by solid papillary architecture lacking myoepithelial cell lining, showing infiltrative, map-like growth patterns. Tumor cells often display plasmacytoid or mucin-rich features, with delicate fibrovascular cores, associated stromal desmoplasia or fat infiltration, and expression of neuroendocrine markers (6).

This study reports the case of a 53-year-old woman who was diagnosed with *in situ* solid papillary carcinoma (SPC) of the breast eight years ago. Five years later, a pulmonary nodule was detected and subsequently confirmed as metastatic infiltrative solid papillary carcinoma (ISPC) of the lung. This case illustrates the morphological spectrum from *in situ* SPC to pulmonary metastatic ISPC, and provides insight into the clinicopathological features of this disease. It also offers valuable information for differential diagnosis and clinical management.

## Case

A 53-year-old perimenopausal female patient underwent a left mastectomy. The pathological diagnosis was intraductal solid papillary carcinoma (SPC) of the breast, with a lesion measuring 0.3 cm in diameter. Immunohistochemical analysis revealed the following results: ER (estrogen receptor): >80%, strong), PR (40%, strong), HER2 (0), Ki67 (15%), synaptophysin (SYN, positive), calponin (positive for myoepithelial cells), and CK5/6 (negative). No further treatment was administered after surgery.

**Figure 1:**
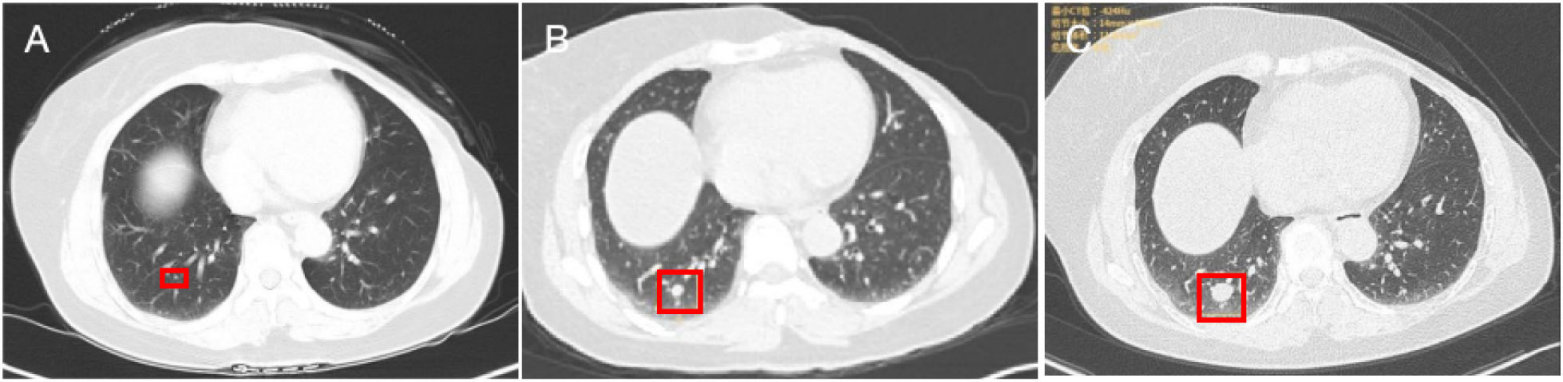
Imaging results. **(A)** A small nodule in the lower lobe of the right lung, marked with a red box. **(B)** The nodule in the lower lobe of the right lung (Se201: Img40), size: 4X4 mm, marked with a red box. **(C)** A solid nodule in the lower lobe of the right lung (Se2: IM40), size: 15X13 mm, marked with a red box. Multiple small solid nodules are also seen in both lungs, with a long diameter range of approximately 3-4 mm. No definite enhancement was observed on the enhanced scan. No enlargement of the hilum in both lungs, and no obvious abnormal nodular shadows in the mediastinum. No thickening of both pleurae, and no significant abnormal density shadows in both pleural cavities.

Five years post-surgury, a routine chest CT scan revealed a tiny nodule in the right lower lobe of the lung, measuring approximately 1 mm in diameter (**Figure 1A**).

Six years and three months post-surgery, follow-up imaging showed that the nodule in the right lower lobe of the lung had increased in size, measuring 4x4 mm (**Figure 1B**).

**Figure 2:**
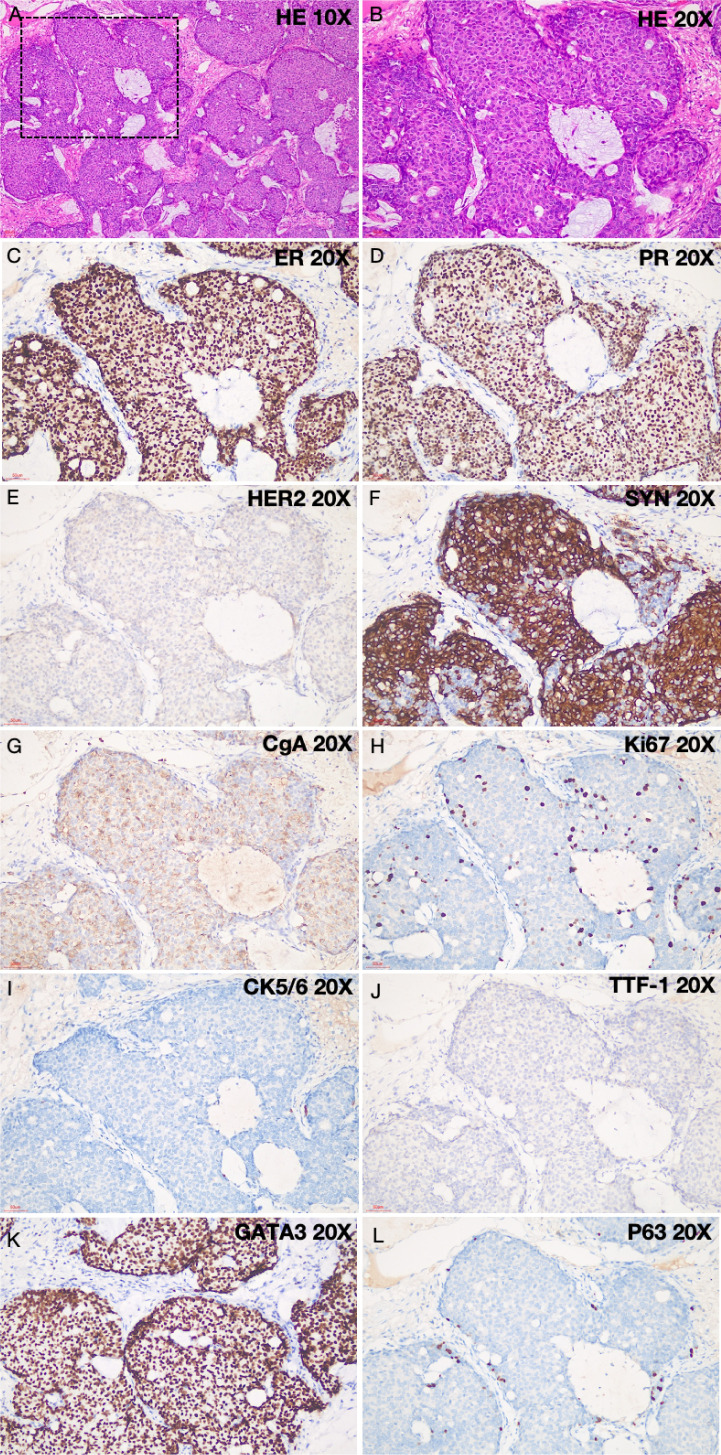
Histological results of tumor biopsy. **(A, B)** Hematoxylin and eosin (HE) stained sections observed under ×10 and ×20 magnifications, respectively. **(A)** Low magnification view shows solid papillary carcinoma with a solid papillary growth pattern. **(B)** High magnification view shows tumor cells that are small to medium-sized, with a round or oval shape. The tumor cell nuclei are graded as intermediate (Grade II), with some cytoplasm showing eosinophilia or pale granular staining. Intracellular and extracellular mucus is observed. **(C)** Estrogen receptor positive immunohistochemical staining; **(D)** Progesterone receptor positive immunohistochemical staining; **(E)** HER2 negative; **(F)** Neuron-specific enolase positive; **(G)** Chromogranin A positive, indicating neuroendocrine differentiation of the tumor; **(H)** Ki67 (15%); **(I)** CK5/6 negative, indicating molecular subtype Luminal A with low tumor malignancy; **(J)** TTF-1 negative, indicating the tumor is not of pulmonary origin but a metastatic tumor; **(K)** GATA3 positive, indicating the tumor is of breast origin; **(L)** P63 negtive.

Seven years and eight months post-surgery, a chest CT scan revealed a solid nodule in the outer basal segment of the right lower lobe, measuring 15x13 mm (**Figure 1C**), which had significantly increased in size compared to previous scans. Additionally, multiple small solid nodules, ranging in size from 3-4 mm in long diameter, were noted in both lungs. No definite enhancement was observed on contrast-enhanced scanning. A thorough physical examination detected no other abnormalities. The patient also denied a history of infectious diseases or other genetic disorders. Given the interval growth of the nodule, a lung tumor resection was subsequently performed. Preoperative tests, including complete blood count, coagulation, liver and kidney function, and tumor markers, showed no significant abnormalities. Postoperative pathological analysis revealed a mass with ill-defined borders, a solid gray-white appearance, and dimensions of 1.5x1.3x0.8 cm. Microscopic examination showed that the tumor tissue formed solid nodules with a fibrous vascular axis, and some cellular nests were irregular, with a serrated or map-like infiltrative growth pattern. Both intra- and extracellular mucus was observed. Tumor cells ranged from small to medium in size, with round or oval shapes. The nuclear grade of the tumor cells was intermediate (Grade II). Some cytoplasm stained eosinophilic or faintly granular (**Figures 2A, B**). Immunohistochemical results (**Figures 2C–L**) were as follows: ER: 90%, strong positive (**Figure 2C**), PR (progesterone receptor): 80%, strong positive, HER2: 0 (negative), Ki-67: 15% positive, P63: negative, CgA (chromogranin A): positive, SYN: positive. These findings suggest the tumor has neuroendocrine differentiation. CK5/6 (cytokeratin 5/6): negative, TTF-1 (thyroid transcription factor-1): negative, GATA3: positive, excluding primary lung cancer and supporting breast origin. Considering the immunohistochemical results, the pathological diagnosis confirmed that the tumor is a metastatic breast invasive ductal carcinoma (ISPC) to the lungs. The tumor is of Luminal A subtype. Following the lobectomy, the patient received antibiotic treatment (latamoxef sodium, later switched to cefoperazone-sulbactam). The surgical incision healed well. Approximately 40 days after surgery, a PET-CT scan showed no abnormalities. The specialist recommended regular follow-up without additional antitumor drug therapy. The patient is currently alive and in good condition.

## Discussion

SPC is a tumor that originates in the breast and exhibits a solid growth pattern. Morphologically, it is characterized by round, well-defined nodules and is considered to have low malignant potential. SPC is relatively rare in breast cancer, primarily affecting postmenopausal elderly women, and accounts for only 1%-2% of primary breast cancers in Asian populations (7, 8). SPC typically presents as a solid, indolent mass, and in some cases, patients may exhibit bloody nipple discharge (9). The metastatic potential of SPC is relatively low, and the primary treatment is surgical excision of the mass. Compared to other types of breast tumors, SPC generally does not require further treatment with radiation or chemotherapy, and the prognosis is relatively favorable.

This patient is a perimenopausal woman, which places her in the high-risk group for SPC. Existing literature suggests that, prior to metastasis, *in situ* SPC is often accompanied by invasive cancer components or progresses to ISPC due to the loss of myoepithelial cells. However, in this case, the patient was diagnosed with *in situ* SPC after undergoing a mastectomy. Eight years post-surgery, ISPC lung metastasis was detected, but previous medical examinations did not show any evidence of invasive tumor (with myoepithelial marker Calponin being positive). The intermediate steps in the process from *in situ* SPC excision to the development of ISPC lung metastasis are still unclear. Typically, for tumor cells to achieve distant metastasis and successfully colonize a distant site, they must first escape from the primary lesion. However, most tumor cells struggle to adapt to the various unfavorable factors encountered during the metastatic process. Only a small fraction of tumor cells are able to survive in the bloodstream as circulating tumor cells (CTCs) (10, 11). During hematogenous dissemination, CTCs interact directly or indirectly with various hematopoietic cells, altering their molecular phenotype to manipulate host cell functions. This enables CTCs to adapt to the circulatory environment and facilitates their extravasation into distant tissues (12, 13). However, detecting CTCs in the early stages of breast cancer is highly challenging due to their very low abundance in the blood. A retrospective study involving over 2,000 breast cancer patients showed that the median number of CTCs in 7.5 mL of blood was fewer than five. This low concentration makes it difficult to reliably detect CTCs, further complicating their use for early detection and monitoring of metastasis in clinical practice (14). In addition, CTC detection in clinical practice is primarily used to assess the effectiveness of radiotherapy and chemotherapy. For tumors where surgical resection is the main treatment approach, CTC testing is generally not performed routinely. Based on this context, we propose a plausible hypothesis that tumor cells from *in situ* SPC may enter and persist in the bloodstream in an occult manner. The lungs, as a “filter” for systemic venous return, are often the first organ to which tumor cells arrive during hematogenous metastasis. Due to the small diameter of pulmonary capillaries (approximately 5-15 µm), larger tumor cells are prone to become trapped in these vessels, where they can form metastatic foci. Additionally, the rich blood supply to lung tissue provides ample nutritional support for the metastatic cancer cells, facilitating their growth and survival (15–17). Although clinical reports on ISPC are relatively limited, studies have indicated that, in addition to local axillary lymph node metastasis, the primary site of distant metastasis for ISPC is the lungs (6, 18, 19). The distant metastasis pattern observed in this case further supports this finding, suggesting that SPC, even at the *in situ* stage, may possess a highly concealed metastatic potential. This highlights the importance of vigilant long-term monitoring for metastasis, even in cases where the tumor appears localized. It should be emphasized, however, that the underlying mechanism proposed here remains speculative and warrants validation through dedicated studies focused on the detection and molecular characterization of CTCs in SPC patients.

A previous follow-up study on SPC spanning 30 years showed that the average Ki-67 index for SPC with associated invasive cancer was 6.3%. This relatively low proliferation rate suggests that SPC, in its early or localized form, tends to have a slower growth pattern compared to other more aggressive forms of cancer, which may be one of the reasons for its generally favorable prognosis when detected early. In this case, both the *in situ* lesion and the lung metastatic lesion had a Ki-67 index of 15%, which, although slightly higher than the average, still falls within the low expression range. This indicates that the proliferative activity of SPC itself is relatively low. Furthermore, the consistency of the Ki-67 index between the primary lesion and the metastatic lesion suggests that there was no significant increase in proliferative capacity during the metastatic process. Additionally, studies have confirmed that all steroid hormone receptors are expressed in lung tissue, and locally produced estradiol can activate ER-α through a paracrine mechanism, potentially influencing the behavior of metastatic cells in the lung (20–22). The upregulation of ER/PR in the lung metastatic lesion in this case suggests that the proliferation of ISPC may be more driven by endogenous endocrine signals, which could reactivate tumor cells that had been in a dormant state for an extended period. Therefore, we recommend longer term follow up after SPC surgery. This approach would help in the early identification of potential recurrence or metastatic risks, allowing for timely intervention and management.

From a histological perspective, the pulmonary nodular lesion in this case presented as an expansile invasive solid papillary carcinoma. Myoepithelial cells within and surrounding the tumor nodules showed negative P63 staining, and the invasive cells had breached the basement membrane, indicating metastatic potential. Therefore, it was regarded as invasive carcinoma and should be treated and evaluated according to the principles for invasive cancer. Previously, the patient’s primary lesion had been diagnosed as *in situ* SPC, wherein myoepithelial cells (positive for calponin) were observed within the fibrovascular cores of some solid papillae and around the tumor nests. This form is considered a variant of high-grade ductal carcinoma *in situ*, in which cancer cells are confined to the ductal structures without breaching the basement membrane or invading the surrounding stroma, thus classifying it as non-invasive. Based on the initial diagnosis of carcinoma *in situ*, no further adjuvant therapy was administered postoperatively.

It is noteworthy that the pulmonary nodular lesion in this case retained a solid papillary architecture, which may morphologically resemble primary pulmonary neuroendocrine tumors, such as carcinoid tumors or neuroendocrine carcinomas. The latter typically exhibit uniform cellular morphology composed mainly of round or oval-shaped cells, and express neuroendocrine markers. However, neuroendocrine tumors lack papillary structures and mucus secretion, and they do not express estrogen or progesterone receptors, which allows them to be differentiated from SPC. Additionally, previous studies have emphasized the need to distinguish SPC from other invasive breast cancers to avoid overtreatment, as SPC typically has a more indolent course and lower metastatic potential (23). Common invasive breast cancers include invasive ductal carcinoma (IDC), invasive lobular carcinoma (ILC), mucinous carcinoma, and invasive papillary carcinoma. IDC lacks the papillary structure with a fibrous vascular axis and typically does not express neuroendocrine markers, with molecular subtypes being diverse. ILC often presents with a lobular *in situ* carcinoma morphology and a loss of cell adhesion, with some cases exhibiting a histiocytic appearance. E-cadherin is usually negative, while GATA3 is positive. When extracellular mucus production is present, it needs to be differentiated from solid papillary carcinoma (ISPC). ILC does not have a papillary structure and does not express neuroendocrine markers. Mucinous carcinoma, especially the cellular type, may be difficult to distinguish from ISPC when there is significant intracellular and extracellular mucus. However, mucinous carcinoma generally lacks solid papillary structures, does not show neuroendocrine differentiation, and is commonly classified as Luminal B type in molecular subtyping. Invasive papillary carcinoma features complex branching papillary structures with a fibrous vascular axis at the core of the papilla. Tumor cells are predominantly columnar, with eosinophilic cytoplasm and light staining. Immunohistochemistry typically does not express neuroendocrine markers, which can help differentiate it from ISPC.

This study reports a postmenopausal female patient who was diagnosed with *in situ* SPC of the breast 8 years ago. After surgery, no further treatment was administered, and 5 years later, she developed ISPC lung metastasis. Although SPC is not an uncommon entity, metastasis from non-invasive SPC is exceedingly rare—a key point that merits emphasis. This report elaborates on the distinct histopathological characteristics of both *in situ* and invasive SPC and offers a preliminary discussion of their potential metastatic mechanisms. Based on this case, we recommend that patients with SPC undergo more frequent and long-term follow-up postoperative surveillance, particularly within the early years following surgery. Close monitoring of the patient’s condition is essential for the timely detection of potential recurrence or metastasis. However, due to the exceptionally low incidence of SPC, especially metastatic cases originating from non-invasive lesions, the number of reported cases in the literature is limited. Numerous questions persist regarding precise pathological classification, optimal clinical management, and the underlying mechanisms of invasion and metastasis. Further multidisciplinary studies are essential to improve the understanding of this disease and to refine diagnostic and therapeutic strategies.

## Data Availability

The original contributions presented in the study are included in the article/supplementary material. Further inquiries can be directed to the corresponding author.
